# Supplementary material for: Enhanced top-down sensorimotor processing in somatic anxiety
Source: Transl Psychiatry. 2022 Jul 25;12:295. doi: 10.1038/s41398-022-02061-2 (PMC9314421; doi:10.1038/s41398-022-02061-2)
Supplement: Supplementary file 2 — Supplementary figure legends [file 41398_2022_2061_MOESM2_ESM.docx]

**Supplementary figure legends**

Supplementary figure 1: Group mean connectivity values (columns: from, rows: to)

Supplementary figure 2: FSA connectivity values (columns: from, rows: to)

Supplementary figure 3: FA connectivity values (columns: from, rows: to)

Supplementary figure 4: SEX connectivity values (columns: from, rows: to)

Supplementary figure 5: AGE connectivity values (columns: from, rows: to)

Supplementary figure 6: Frequency distribution of Fear-Somatic Arousal Score before and after elimination of 74 participants
